# Assessment of Physicochemical Characteristics and Microbiological Quality in Broiler Chicken Breast Muscle (*Pectoralis major*) Subjected to Different Temperatures and Lengths of Cold Transportation

**DOI:** 10.3390/foods10040874

**Published:** 2021-04-16

**Authors:** Muhammad Nizam Hayat, Ubedullah Kaka, Awis Qurni Sazili

**Affiliations:** 1 Department of Animal Science, Faculty of Agriculture, Universiti Putra Malaysia, Serdang 43400, Selangor, Malaysia; nizamhayat88@yahoo.com.my; 2 Department of Companion Animal Medicine and Surgery, Faculty of Veterinary Medicine, Universiti Putra Malaysia, Serdang 43400, Selangor, Malaysia; dr_ubedkaka@upm.edu.my; 3 Institute of Tropical Agriculture and Food Security, Universiti Putra Malaysia, Serdang 43400, Selangor, Malaysia; 4 Halal Products Research Institute, Putra Infoport, Universiti Putra Malaysia, Serdang 43400, Selangor, Malaysia

**Keywords:** broiler, transportation temperature, transportation duration, pectoralis major, meat quality, coliform, *Salmonella*, cold truck

## Abstract

Cold truck transportation is considered one of the most integral parts in a food processing chain. However, countless cases of product spoilage and food poisoning incidents have proven that temperature control during transport has been neglected. Literature on the impact of temperature during distribution is scarce. The objective of this study was to investigate the impact of various transportation temperatures and travel duration on the meat quality and microbial population of broiler chicken breast muscle. Sixty broiler chickens (42 days old) were slaughtered and eviscerated; they then had their breast muscles removed (each bird provided two breast muscle samples: left breast and right breast), which were wrapped in plastic film. All 120 packed boneless breasts (PBBs) were then placed at −18 °C for 24 h. After 24 h, the 60 PBB samples were subjected to transportation for 1 h at 4 °C (20 PBBs), 10 °C (20 PBBs), and 15 °C (20 PBBs) while the remaining 60 samples were transported for 5 h at 4 °C (20 PBBs), 10 °C (20 PBBs), and 15 °C (20 PBBs) before analyses. The samples transported at higher temperatures exhibited higher populations of coliform and *Salmonella* than those transported at lower temperatures. A significant impact of the transportation duration on the *Salmonella* population was only observed in samples transported at 4 °C for 5 h. However, a significant impact of transportation temperature on color was only recorded for the redness (a*) values, where the samples transported at higher temperatures exhibited higher redness (a*) values. Significant increases in lightness (L*) and yellowness (b*) values as well as decreases in redness (a*) and pH values were recorded in samples subjected to longer durations of transportation across all the temperatures observed in this study.

## 1. Introduction

Cold truck transportation plays a pivotal role in a food processing chain and in determining the final meat quality before the meat reaches the consumers. Investigating cold transportation is a continuing concern within the meat industry. For the last few decades, a growing trend toward an improvement in meat quality, especially in transportation technology, has been noticed, mainly due to the fluctuation in demand for meat products. The poultry industry in Malaysia has grown significantly during the last few decades due to the extent of exportation of poultry products to countries such as Brunei, Papua New Guinea, Vietnam, China, Singapore, Hong Kong, and Japan.

A longer delivery distance leads to greater concerns among consumers regarding meat quality and food safety, which may be compromised along the supply chain. Previous research has established that the spoilage of food products is a result of a combination of several factors such as processing, transportation, and storage in the market [1,2,3,4]. For instance, commonly found spoilage microorganisms, such as lactic acid bacteria, coliforms, *Pseudomonas*, *Brochothrix thermosphacta, Salmonella,* and yeasts [5,6,7,8,9,10,11], can contaminate food products along the processing chain if no necessary hygiene preventive measures are carried out. Therefore, a system to prevent health hazards caused by spoilage during processing, known as the Hazard Analysis Critical Control Point (HACCP) system [12], was created. HACCP is a systematic approach to control any chemical, physical, or microbiological hazards that may arise in a food-processing facility or food supply chain. This system provides guidelines for the systematic management of meat products, including raw material selection and control of conditions during processing and distribution.

Previous observations have indicated serious deviations in the execution of the system, especially during transportation, due to a lack of direct control by the manufacturer [13,14,15]. Most of the time, temperature control is lacking between the processing plant and the distributor’s domestic storage. Although most meat products are transported using a cold truck, the temperature is not monitored. Most processing plants try to reduce the total processing cost, including transportation cost, by minimizing the amount of fuel consumption during delivery. This can be achieved by turning off the air-conditioning system that was supposed to control the temperature of the poultry meat during transportation. A much-debated question is whether the industry should simply accept the undesirable increase in production cost by retaining the current price, thus reducing their profit and quality, or whether they should raise the price to maintain their profitability and, most importantly, to ensure end product quality and safety. To date, little attention has been paid to this problem despite it being a major crisis in almost all processing plants [16]. Thus far, most studies conducted have focused on the optimal temperature for storage [3,4,13,14,15,17,18,19,20,21]. No studies have been reported on the impact of cold transportation temperature on meat quality in Malaysia. Most recently, Ab Aziz et al. [5] reported significant effects of the temperature and duration of storage on poultry meat quality and microbial growth. However, the main concern that needs to be addressed currently is the impact of varying temperatures and travel durations on meat quality and microbiological spoilage, which have a huge influence on the meat’s physicochemical properties and the consumer’s wellbeing. Temperature is not only a major issue during storage but also a major issue during transit from the processing plant to the outlet due to the possibility of problems with the air-conditioning system or mishandling by the workers during transportation. Therefore, this study was conducted to assess the impact of different transportation temperatures and durations on meat quality characteristics and microbial populations.

## 2. Materials and Methods

In this study, 60 broiler chickens (42 days old, Cobb500) were randomly selected from 500 chickens slaughtered in a commercial poultry processing plant as per the protocols and standards outlined in [22]. Following evisceration, the dressed carcasses were deboned and trimmed (each bird provided two breast muscle samples: left and right sides of chicken breast). Before transportation, all 120 packed boneless breasts (PBBs) were stored at −18 °C for 24 h. After storage for 24 h, 60 samples of the PBBs were loaded into the cold truck and transported for 1 h at 4 °C (20 PBBs), 10 °C (20 PBBs), and 15 °C (20 PBBs), while the remaining 60 samples were transported for a duration of 5 h at 4 °C (20 PBBs), 10 °C (20 PBBs), and 15 °C (20 PBBs). Breast samples were then transported for subsequent analyses to the Meat Science Laboratory, Department of Animal Science, Faculty of Agriculture, Universiti Putra Malaysia.

### 2.1. Meat Quality Analysis

#### 2.1.1. Drip Loss and Cooking Loss Determination

Drip loss was measured for each pectoralis major muscle sample at the end of 1 and 5 h of transportation, whereby 30 g of the pectoralis major sample was weighed and placed into polyethylene bags, which were subsequently sealed and vacuumed. The meat samples were taken out from the bag and reweighed after 24 h storage at 4 °C. The following formula [23] was used in order to determine the percentage drip loss:

$$Drip\;loss\;\left(\%\right)=\left(\frac{\left(Wa-Wb\right)}{Wa}\right)\times 100,$$

whereby *Wa* is the muscle sample weight before storage (g) and *Wb* is the muscle sample weight after storage (g).

Determination of cooking loss was conducted on each pectoralis major sample at the end of 1 and 5 h of transportation. Approximately 30 g of each meat sample was placed in a polyethylene bag and submerged in a water bath preset at 80 °C until the internal temperature reached 78 °C. Upon reaching the preferred internal temperature, the samples were allowed to cook for another 10 min [24]. The bags containing samples were then placed under running tap water for 30 min to cool down, before being dabbed dry using paper towels without squeezing and reweighed. Cooking loss percentage was then determined using the following equation [23]:

$$Cooking\;loss\;\left(\%\right)=\left(\frac{\left(Wx-Wy\right)}{Wx}\right)\times 100,$$

where *Wx* is the muscle sample weight prior to cooking in the water bath (g) and *Wy* is the muscle sample weight after cooking in the water bath (g).

#### 2.1.2. Color Value Measurement

Meat color characteristics (L*: lightness, a*: redness, and b*: yellowness) were measured using the ColorFlex^®^ system [25] with an illuminant D65 as the light source and a 10° standard observer (aperture size of 5 cm). The instrument was calibrated against a white and black reference tile prior to use. The L*, a*, and b* color coordinate values were measured on the cut surface of the muscle after a 30 min bloom time at 4 °C. Three measurements were taken from each meat sample at the end of each transportation duration.

#### 2.1.3. Shear Force Measurement 

After each transportation duration (1 and 5 h), the meat samples were thawed at 4 °C, placed in an aluminum pan, and covered with aluminum foil. The cuts were then cooked in a water bath to an internal temperature of 78 °C and maintained at this temperature for an additional 10 min for further cooking. After cooking, the cuts were chilled overnight at 4 °C. From each cooked sample, at least three replicate blocks (1 × 1 × 2 cm) were cut parallel to the direction of the muscle fibers. Each block was sheared perpendicularly in the longitudinal direction of the fibers with a Volodkevich bite jaw (stainless-steel probe shaped like an incisor) after being placed on the base plate of a TA-HD plus texture analyzer (Stable Micro System, Surrey, UK).

#### 2.1.4. pH Value Determination

The pH of each meat sample was determined upon completion of the 1 h and 5 h transportation, according to the method described by [26]. The indirect pH determination of pectoralis major muscles was carried out using a portable pH meter (Mettler Toledo, AG 8603, Switzerland). The pH meter was first calibrated at pH 4.0 and then 7.0 prior to use. Approximately 0.5 g of each crushed muscle sample was homogenized (Wiggen Hauser^®^ D-500, Germany) for 20 s in 10 mL of ice-cold double-distilled water (ddH2O) in the presence of 5 mM sodium iodoacetate (Merck Schuchardt OHG, Germany) to prevent further glycolysis [26]. The handheld glass electrode attached to the pH meter was used to measure the pH of the resultant homogenates.

### 2.2. Microbiological Analysis

First, 5 g of the subsample was aseptically dissected from each meat sample upon completion of 1 h and 5 h transportation, weighed, and transferred into a Stomacher bag containing 45 mL of 2.25% peptone water (Merk KGaA, Germany) and homogenized using a Stomacher (Inter Science, France) for 120 s at room temperature [27]. For microbial enumeration, 100 µL samples of a 10-fold dilution in peptone water were spread on the surface of dry media. Ten-fold dilutions were spread on Petri dishes in duplicate for enumerations of coliform on a McConkey agar (Merk KGgA, Germany) and *Salmonella* on a Xylose Lysine Deoxycolate (XLD) agar (Merk KGgA, Germany). For all bacterial counts, the plates were incubated at 37 °C for 24 h.

### 2.3. Design and Statistical Analysis

The experiment adopted a completely randomized design (CRD). All data were analyzed by two-way analysis of variance (ANOVA) using the Statistical Analysis System software package, version 9.3 (SAS Institute Inc., Cary, NC, USA). The differences between means were calculated using Duncan’s new multiple range test.

## 3. Results

### 3.1. Drip Loss and Cooking Loss

Table 1 and Table 2 present the differences in drip loss and cooking loss percentages of pectoralis major muscles subjected to different transportation temperatures and durations, respectively. There was no significant effect of the interaction between transportation temperature and duration on drip loss in the meat samples. There were also no significant differences (*p* > 0.05) in drip loss observed between the meat samples transported at different temperatures for both 1 h and 5 h transport durations. Likewise, transportation duration had no significant effect on meat samples, with the exception of samples transported at 10 °C (*p* = 0.025) (Table 1).

As presented in Table 2, there was no significant interaction (*p* > 0.05) observed between transportation temperatures and duration in their effects on the cooking loss percentage. In this study, neither the transportation temperature nor duration had any effect on the cooking loss of meat samples.

### 3.2. Color Values

Table 3 shows the results of three color characteristics, lightness (L*), redness (a*), and yellowness (b*), for pectoralis major muscles transported for different transportation durations and at different temperatures. In this study, there was no significant interaction between transportation temperature and duration detected for all color characteristics (L*, a*, and b*). The lightness values were not affected by transportation temperature; however, the values were affected significantly (*p* < 0.05) by transportation duration. Meat samples subjected to a transportation duration of 5 h presented a higher L* value (*p* < 0.05) than those transported for 1 h duration at 4 °C, 10 °C, and 15 °C (Table 3).

The redness (a*) value was significantly affected (*p* < 0.05) by both transportation temperature and duration. Meat samples transported for the shorter duration (1 h) exhibited a significantly higher (*p* < 0.05) redness (a*) value than samples transported for a longer duration (5 h). There was no significant difference observed in the redness values between meat samples transported at 4 °C and 10 °C for both 1 h and 5 h of transportation duration. However, the redness values were higher for the meat samples transported at 15 °C (Table 3). On the other hand, yellowness (b*) values were significantly affected (*p* < 0.05) by transportation duration, while transportation temperature had no significant effects on b* value. Meat samples transported for a shorter duration (1 h) had significantly higher b* values than samples transported for a longer duration (5 h).

### 3.3. pH Values

Table 4 presents the differences in pH of pectoralis major muscle samples after being transported at different temperatures and for different durations. According to the results, there was no significant effect observed for the interaction between transportation temperature and duration on pH value. It was also observed that pH value was not significantly affected by transportation temperature, regardless of transportation duration. On the contrary, pH value was significantly affected (*p* < 0.05) by transportation duration. The observation showed that, with an increase in transportation duration, pH value significantly decreased (*p* < 0.05) for all transportation temperatures. In summary, these results show that, with an increase in the transportation duration, the pH of the meat samples decreased gradually (Table 4).

### 3.4. Shear Force Values

The results for the shear force values (tenderness) of pectoralis major muscle samples transported at different temperatures and for different durations are presented in Table 5. No significant effect of the interaction between transportation temperature and duration was observed on the shear force value (Table 5). Furthermore, the shear force value was not significantly affected by the storage temperature or duration.

### 3.5. Coliform and Salmonella Population

The effects of the transportation temperature, the duration, and their combination on coliform and *Salmonella* populations are presented in Table 6 and Table 7, respectively. No significant effect of the interaction between transportation temperature and duration on the populations of both coliform and *Salmonella* was observed. However, the coliform population was significantly affected (*p* < 0.05) by transportation temperature regardless of transportation duration (1 h and 5 h). It was observed that meat samples transported at 4 °C recorded a significantly lower (*p* < 0.05) coliform population compared to samples transported at 10 °C and 15 °C. The population of coliform for meat samples transported at 4 °C and 10 °C were found to be significantly affected (*p* < 0.05) by the changes in transportation duration (1 and 5 h). The population of coliform increased significantly at 4 °C and 10 °C after being transported for a longer duration compared to meat samples transported for a shorter duration.

*Salmonella* populations were significantly affected (*p* < 0.05) by transportation temperatures. As presented in Table 7, *Salmonella* populations increased (*p* < 0.05) with an increasing temperature of transportation. However, *Salmonella* populations were only significantly (*p* = 0.0131) affected by the transportation duration at 4 °C.

## 4. Discussion

### 4.1. Effects of Different Transportation Temperatures and Transportation Durations on Water Holding Capacity

Fresh meat comprises approximately 75% water. However, the amount of water in meat may change after processing due to drip loss, evaporation, and cooking loss [28]. The results from this study revealed no significant impact of transportation temperature or duration on the water-holding capacity of the meat samples, except for the drip loss percentage in meat samples transported at 10 °C. A similar trend was observed for cooking loss. Previous studies reported similar findings, where they suggested that the changes in water-holding capacity may be the result of water loss through drip loss, evaporation, and cooking loss and that they are affected by several factors such as pH, storage temperature, glycogen content, water crystal formation, and thawing temperature [4,13,17,29]. The drip loss percentages of meat samples are highly dependent on the changes in pH of meat before, during, and after processing [17]. As observed from the present study, differences in drip loss percentage could be caused by a decrease in pH, which resulted in acidification in meat, consequently leading to a weakening of the water-holding capacity. However, it is important to note that the initial glycogen concentration (mg/g) available in muscles prior to slaughtering can also have an effect on the final pH value [30]. This is because glycogen is used as a substitute for free fatty acids and because glucose is used as the energy supplier for muscle metabolism. The end product of glycogen breakdown to supply energy toward muscle metabolism is lactic acid. A higher concentration of glycogen results in increased production of lactic acid, consequently reducing the pH of meat. This is why, at decreased pH, the muscle protein starts denaturing, thus affecting the muscle’s water-binding ability [31].

In this study, higher drip loss was recorded only for muscle samples transported at 10 °C for 1 h compared to muscle samples transported for 5 h. Muscle samples that were stored at −18 °C for 24 h were directly transported using cold trucks. After storage at −18 °C, the water in the extracellular spaces might become frozen. After 24 h of cold storage, the ice in the extracellular spaces melts, thus giving rise to water activity. The water then causes a net flow into the intracellular spaces, where it gets absorbed by dehydrated fiber [17]. In this study, the amount of drip loss was higher in muscle samples transported for 1 h due to the rate at which the dehydrated fiber absorbed the water, exceeded by the rate at which the ice melted. The water that did not gets absorbed by the fiber could be excreted, thus increasing the amount of exudates. Overall, there is still no agreement in the scientific literature on how the temperature during freezing, storage, thawing, and transportation influences the water-holding capacity of meat [17,19,20]. As for cooking loss, the percentages remained unaffected by transportation temperature and duration. This is in agreement with the results documented by Leygonie et al. [17] and Vieira et al. [19], where muscle samples thawed at different rates showed no significant differences. Although no significant results were observed, the cooking loss percentages recorded were numerically higher than those observed for drip loss. The higher cooking loss percentage could possibly be explained by protein denaturation during the cooking process, which, in turn, could cause chemically bound water to be released [19].

### 4.2. Effects of Different Transportation Temperatures and Transportation Durations on Color

The physical appearance of meat is one of the main attributes of palatability. The color of meat is regarded as one of the essential factors for consumers in determining meat quality, freshness, and safety. In this study, it was noted that meat samples transported for 5 h had a higher lightness (L*) value than those transported for 1 h. A previous study showed that freezing and thawing processes could influence the lightness value [32]. As documented by Muela et al. [32], fresh meat samples possessed the highest lightness value compared to meat subjected to the freezing process. The difference in lightness values between meat samples transported for different durations could be explained by the differences in light reflection due to different amounts of thaw drip [14]. The amount of thaw drip produced for meat samples differs as a function of the amount of protein degradation and the shrinkage of myofibrils. The duration for which the meat is exposed to the surrounding temperature could also play an important role in determining the amount of exudates released and absorbed by the muscle fiber [4,17]. Meat that has been exposed for a longer duration has more time to reabsorb the ice that melts from the extracellular spaces, thus reducing light scattering. However, in this study, the increase in light scattering mainly occurred via a pH-related mechanism. A study by Swatland [33] demonstrated that a decrease in pH would lead to an increase in light scattering, thus causing meat to appear pale compared to meat with a higher pH value, which would appear darker. A decrease in pH would cause the negative electrostatic repulsion of myofilaments to be reduced, thus leading to them moving closer together laterally, resulting in an increase in the refractive index in the lateral direction [33].

In this study, meat samples transported for a shorter duration (1 h) had higher redness (a*) values than those assigned to a longer travel duration (5 h). Previous studies suggested that the muscle redness value depends on the concentration of myoglobin in the skeletal muscle [34]. This is supported by Kim et al. [35] who reported that myoglobin content influenced meat lightness and redness values. Furthermore, an increase in light scattering would cause a reduction in the amount of light being absorbed, thus reducing the red appearance in the meat. A higher lightness value indicates a higher amount of exudate, and a poor water-holding capacity could cause light scattering from the meat surface. Swatland [33] suggested that light scattering was caused by differences in the refractive indices of the sarcoplasm and myofibrils. If a larger difference in the refractive indices is produced, meat products appear paler due to a greater amount of light scattering. Moreover, light scattering could result from the contraction of myofilaments.

It was also suggested that a low pH value in meat could stimulate the oxidation of red myoglobin to brown metmyoglobin. Some researchers suggested that the enzymatic system known as metmyoglobin-reducing activity (MRA) regulates the conversion of oxidized myoglobin (metmyoglobin) back to myoglobin. Abdallah et al. [36] reported that the freezing and thawing process could possibly contribute to a loss of MRA activity. As a result, the brown-colored metmyoglobin would not be converted back to the red-colored myoglobin or oxymyoglobin, thus resulting in a lower redness value.

On the other hand, the current study revealed a significant decrease in the yellowness values (*p* < 0.05) of meat samples after being transported for a longer duration. However, due to an insufficient number of studies on the relationship between yellowness and cold transportation, various theories have been suggested in order to explain the underlying mechanisms. For instance, a study reported that yellowness has a positive correlation with muscle pH [21]. On the other hand, Allen et al. [37] reported a negative correlation between yellowness and muscle pH. A study by Muela et al. [32] showed that yellowness was affected by the freezing method, freezing rate, and display duration, whereas the frozen storage duration did not affect yellowness. The findings by Muela et al. [32] are supported by previous research conducted by Zhu and Brewer [38]. Leygonie et al. [39] and Kobayashi et al. [40] also suggested that a reduction in yellowness (b*) value in the meat could result from microbial spoilage (*Pseudomonas*). *Pseudomonas*-associated spoilage produces a yellow-colored slime layer, which was discovered to reduce metmyoglobin to deoxymyoglobin, thereby producing a poorer yellowness value. However, the impact of temperature on yellowness values in meat is yet to be well-documented.

### 4.3. Effects of Different Transportation Temperatures and Transportation Durations on pH

Muscle pH values play a significant role in determining meat quality, whereby the ultimate pH value (pHu) is influenced by glycogen concentration in the muscles [30,41]. The results of the current study demonstrated a decrease in the pH values of meat samples transported for a longer duration. The current findings are in concordance with the results reported by Leygonie et al. [39] and Salwani et al. [41], who found a decrease in meat pH after freezing and thawing compared to before freezing. Denaturation of the buffer protein during freezing could trigger the release of hydrogen ions, thus decreasing the pH [17]. Leygonie et al. [17] also suggested that an increase in the concentration of solutes due to the accumulation of fluids from meat tissue can cause a fall in pH value. As the time of exposure to surrounding temperatures during transportation differed, the activity of the enzyme in resuming glycolysis would be different for each muscle sample.

### 4.4. Effects of Different Transportation Temperatures and Transportation Durations on Tenderness

Tenderness is one of the most important attributes describing meat quality. Out of all the organoleptic traits, a consumer’s acceptance toward meat is mostly influenced by tenderness [30]. In this study, different transportation temperatures and durations did not affect the shear force values. Devine et al. [42] reported that the shear force values did not differ during the initial aging process and that significant differences were only observed after 8 h of postmortem aging. It was also mentioned that meat stored at lower temperature aged faster and was more tender compared to meat stored at higher temperature. However, previous studies also showed that freezing and thawing could cause an increase in meat tenderness [13,15,17,43,44]. Leygonie et al. [17] suggested that the tenderization effects in meat during freezing and thawing are caused by the formation of ice crystals and breakdown of muscle fibers by enzymes. Furthermore, Salwani et al. [41] reported a significant drop in shear force with an increase in the postmortem aging time. In addition to ice crystal formation, Farouk et al. [29] suggested that the decrease in shear force value could be due to the poor myofibrillar protein strength caused by endogenous protease enzymes.

As mentioned previously, the shear force values observed in this study were not affected (*p* > 0.05) by transportation temperature. These data differ from the results obtained by Yu et al. [30], who reported that the tenderness of pre-rigor frozen chicken muscle is affected by thawing temperature. However, the variations in the outcome of meat tenderness after freezing could be explained by the differences in initial glucose and glycogen concentration and the rate of glycolysis, which determine the onset and extent of postmortem proteolysis [1,41,45].

### 4.5. Effects of Different Transportation Temperatures and Transportation Durations on Microbial Population

Microbial spoilage has a huge impact on meat quality. It relates to the shelf life of the meat and the consumer’s safety, which can ultimately lead to a decrease in market value [18,45,46]. In this study, both the coliform and *Salmonella* populations were significantly affected (*p* < 0.05) by transportation temperature. Meat transported at higher temperatures showed higher populations of coliform and *Salmonella*. It is well known that bacteria in the coliform group such as *Escherichia coli* can grow at a high optimal temperature of 37 °C; however, they can still grow at 8 °C and survive at −20 °C of frozen storage [47]. Even though bacteria such as *Escherichia coli* can still multiply at 8 °C, the rate at which they grow or multiply is suppressed by up to 4–6%.

In the present study, the results revealed that transportation duration had a significant effect on coliform and *Salmonella* population only for meat transported at 4 °C. A similar result was also obtained by Nakyinsige et al. [24], who recorded an increase in the population of bacteria with an increase in postmortem storage time at 4 °C. In addition, Vieira et al. [19] recorded a similar trend, where the population of psychotropic bacteria increased with the increased aging time in beef. It is suggested that the solutes released from thawing provide a great medium for microbial growth because of the high protein, vitamin, and mineral content [17].

## 5. Conclusions

The effects of transportation temperature and duration on the physicochemical characteristics and microbiological population of pectoralis major muscles in commercial broiler chickens were independent of each other. However, it was observed that individual effects of transportation temperature and transportation duration were noticeable in some of the meat quality traits. For instance, increased transportation temperature resulted in a higher redness value and increased microbial population. On the other hand, a longer transportation duration (at 10 °C) decreased drip loss and muscle pH values. Similarly, all color parameters (L, a*, and b*) were affected by transportation duration, with increased lightness but decreased redness and yellowness values observed as transportation duration increased. In addition, a longer transport duration resulted in an increase in both coliform and *Salmonella* populations in the meat samples.

## Figures and Tables

**Table 1:** Effects of transportation temperatures (4 °C, 10 °C, and 15 °C) for two different transportation durations (1 and 5 h) on drip loss (%) of the pectoralis major muscles in broiler chickens.

Temperature (°C)	Transportation Duration		*p*-Value (Dur)	*p*-Value (Temp × Dur)
	1 h	5 h		
	Mean	Mean		
4	2.17 ± 0.22 ^a,x^	1.93 ± 0.12 ^a,x^	0.3755	0.3926
10	2.39 ± 0.18 ^a,x^	1.64 ± 0.11 ^a,y^	0.0254	
15	2.27 ± 0.32 ^a,x^	1.68 ± 0.09 ^a,x^	0.0906	
*p*-Value (Temp)	0.8365	0.1797		

^a^ Means with identical superscript letters within the same column did not differ significantly (*p* > 0.05) across transportation temperatures. ^x,y^ Means with different superscript letters within the same row differ significantly (*p* < 0.05) across transportation durations. Means are presented as ± SEM (standard error of the mean). Temp: temperature; Dur: duration.

**Table 2:** Effects of transportation temperatures (4 °C, 10 °C, and 15 °C) for two different transportation durations (1 and 5 h) on cooking loss (%) of pectoralis major muscles in broiler chickens.

Temperature (°C)	Transportation Duration		*p*-Value (Dur)	*p*-Value (Temp × Dur)
	1 h	5 h		
	Mean	Mean		
4	4.01 ± 0.15 ^a,x^	3.89 ± 0.12 ^a,x^	0.5532	0.9695
10	3.65 ± 0.23 ^a,x^	3.88 ± 0.13 ^a,x^	0.3641	
15	4.04 ± 0.19 ^a,x^	3.86 ± 0.12 ^a,x^	0.4534	
*p*-Value (Temp)	0.3412	0.9888		

^a^ Means with identical superscript letters within the same column did not differ significantly (*p* *>* 0.05) across transportation temperatures. ^x^ Means with identical superscript letters within the same row did not differ significantly (*p* < 0.05) across transportation durations. Means are presented as ± SEM (standard error of the mean). Temp: temperature; Dur: duration.

**Table 3:** Effects of transportation temperatures (4 °C, 10 °C, and 15 °C) for two different transportation durations (1 and 5 h) on color characteristics (lightness (L*), redness (a*), and yellowness (b*)) of pectoralis major muscles in broiler chickens.

Color	Temperature (°C)	Transportation Duration		*p*-Value (Dur)	*p*-Value (Temp × Dur)
		1 h	5 h		
		Mean	Mean		
Lightness (L*)	4	49.62 ± 0.71 ^a,y^	56.74 ± 0.85 ^a,x^	0.0002	0.0619
	10	48.09 ± 0.35 ^a,y^	57.15 ± 0.35 ^a,x^	<0.0001	
	15	48.16 ± 0.47 ^a,y^	57.37 ± 0.52 ^a,x^	<0.0001	
	*p*-Value (Temp)	0.1142	0.7609		
Redness (a*)	4	3.76 ± 0.19 ^b,x^	2.69 ± 0.20 ^b,y^	0.0052	0.9467
	10	3.72 ± 0.07 ^b,x^	2.55 ± 0.09 ^b,y^	<0.0001	
	15	4.52 ± 0.09 ^a,x^	3.47 ± 0.12 ^a,y^	0.0002	
	*p*-Value (Temp)	0.0017	0.0015		
Yellowness (b*)	4	16.99 ± 0.24 ^a,x^	12.93 ± 0.53 ^a,y^	0.0001	0.9253
	10	17.19 ± 0.17 ^a,x^	13.55 ± 0.19 ^b,y^	<0.0001	
	15	17.18 ± 0.18 ^a,x^	13.03 ± 0.36 ^a,y^	<0.0001	
	*p*-Value (Temp)	0.7327	0.4819		

^a,b^ Means with different superscript letters within the same column differ significantly (*p <* 0.05) across transportation temperatures. ^x,y^ Means with different superscript letters within the same row differ significantly (*p <* 0.05) across transportation durations. Means are presented as ± SEM (standard error of the mean). Temp: temperature; Dur: duration.

**Table 4:** Effects of transportation temperatures (4 °C, 10 °C, and 15 °C) for two different transportation durations (1 and 5 h) on the pH values of pectoralis major muscles in broiler chickens.

Temperature (°C)	Transportation Duration			*p*-Value (Dur)	*p*-Value (Temp × Dur)
	0 h	1 h	5 h		
	Mean	Mean	Mean		
4	5.68 ± 0.07 ^a,x^	5.27 ± 0.07 ^a,y^	5.11 ± 0.05 ^a,y^	0.0001	
10	5.65 ± 0.09 ^a,x^	5.17 ± 0.07 ^a,y^	5.07 ± 0.05 ^a,y^	0.0002	0.8273
15	5.77 ± 0.02 ^a,x^	5.26 ± 0.07 ^a,y^	5.14 ± 0.07 ^a,y^	<0.0001	
*p*-Value (Temp)	0.3997	0.5369	0.7763		

^a^ Means with identical superscript letters within the same column did not differ significantly (*p* *>* 0.05) across transportation temperatures. ^x,y^ Means with different superscript letters within the same row differ significantly (*p <* 0.05) across transportation durations. Means are presented as ± SEM (standard error of the mean). Temp: temperature; Dur: duration.

**Table 5:** Effects of transportation temperatures (4 °C, 10 °C, and 15 °C) for two different transportation durations (1 and 5 h) on the shear force values (kg) of pectoralis major muscles in broiler chickens.

Temperature (°C)	Transportation Duration			*p*-Value (Dur)	*p*-Value (Temp × Dur)
	0 h	1 h	5 h		
	Mean	Mean	Mean		
4	1.60 ± 0.02 ^a,x^	1.56 ± 0.02 ^a,x,y^	1.53 ± 0.02 ^a,y^	0.0696	
10	1.57 ± 0.04 ^a,x^	1.54 ± 0.04 ^a,x^	1.54 ± 0.06 ^a,x^	0.8749	0.7110
15	1.58 ± 0.02 ^a,x^	1.56 ± 0.02 ^a,x^	1.54 ± 0.03 ^a,x^	0.4182	
*p*-Value (Temp)	0.6992	0.8134	0.9924		

^a^ Means with identical superscript letters within the same column did not differ significantly (*p* *>* 0.05) across transportation temperatures. ^x,y^ Means with different superscript letters within the same row differ significantly (*p <* 0.05) across transportation durations. Means are presented as ± SEM (standard error of the mean). Temp: temperature; Dur: duration.

**Table 6:** Effects of transportation temperatures (4 °C, 10 °C, and 15 °C) for two different transportation durations (1 and 5 h) on the coliform population (log_10_) of pectoralis major muscles in broiler chickens.

Temperature (°C)	Transportation Duration		*p*-Value (Dur)	*p*-Value (Temp × Dur)
	1 h	5 h		
	Mean	Mean		
4	5.71 ± 0.02 ^b,y^	5.95 ± 0.02 ^b,x^	<0.0001	
10	5.91 ± 0.05 ^a,y^	6.08 ± 0.04 ^a,x^	0.0275	0.0526
15	6.02 ± 0.05 ^a,x^	6.13 ± 0.01 ^a,x^	0.0610	
*p*-Value (Temp)	0.0007	0.0021		

^a,b^ Means with different superscript letters within the same column differ significantly (*p <* 0.05) across transportation temperatures. ^x,y^ Means with different superscript letters within the same row differ significantly (*p <* 0.05) across transportation durations. Means are presented as ± SEM (standard error of the mean). Temp: temperature; Dur: duration.

**Table 7:** Effects of transportation temperatures (4 °C, 10 °C, and 15 °C) for two different transportation durations (1 and 5 h) on the *Salmonella* population (log_10_) of pectoralis major muscles in broiler chickens.

Temperature (°C)	Transportation Duration		*p*-Value (Dur)	*p*-Value (Temp × Dur)
	1 h	5 h		
	Mean	Mean		
4	6.53 ± 0.06 ^b,y^	6.74 ± 0.02 ^c,x^	0.0131	
10	6.80 ± 0.07 ^a,y^	6.91 ± 0.04 ^b,x^	0.2219	0.2989
15	6.95 ± 0.08 ^a,x^	7.05 ± 0.02 ^a,x^	0.2355	
*p*-Value (Temp)	0.0040	<0.0001		

^a,b,c^ Means with different superscript letters within the same column differ significantly (*p <* 0.05) across transportation temperatures. ^x,y^ Means with different superscript letters within the same row differ significantly (*p <* 0.05) across transportation durations. Means are presented as ± SEM (standard error of the mean). Temp: temperature; Dur: duration.

## Data Availability

Not applicable.

## References

[1] Adeyemi K.D., Sazili A.Q. (2014). Efficacy of carcass electrical stimulation in meat quality enhancement: A review. Asian-Australas J. Anim. Sci..

[2] McCrea B.A., Tonooka K.H., VanWorth C., Boggs C.L., Atwill E.R., Schrader J.S. (2006). Prevalence of *Campylobacter* and *Salmonella* species on farm, after transport, and at processing in specialty market poultry. Poult Sci..

[3] Devine C.E., Graham R.G., Lovatt S., Chrystall B.B., Jeremiah L.E. (1995). Chapter 2: Red meats. Freezing Effects on Food Quality.

[4] Anon M.C., Calvelo A. (1980). Freezing rate effects on the drip loss of frozen beef. Meat Sci..

[5] Ab Aziz M.F., Hayat M.N., Kaka U., Kamarulzaman N.H., Sazili A.Q. (2020). Physico-Chemical Characteristics and Microbiological Quality of Broiler Chicken *Pectoralis major* Muscle Subjected to Different Storage Temperature and Duration. Foods.

[6] Poghossian A., Geissler H., Schöning M.J. (2019). Rapid methods and sensors for milk quality monitoring and spoilage detection. Biosens. Bioelectron..

[7] Rouger A., Tresse O., Zagorec M. (2017). Bacterial contaminants of poultry meat: Sources, species, and dynamics. Microorganisms.

[8] Trmčić A., Martin N.H., Boor K.J., Wiedmann M. (2015). A standard bacterial isolate set for research on contemporary dairy spoilage. J. Dairy Sci..

[9] Ledenbach L.H., Marshall R.T. (2009). Microbiological spoilage of dairy products. Compendium of the Microbiological Spoilage of Foods and Beverages.

[10] Liu F., Yang R.Q., Li Y.F. (2006). Correlations between growth parameters of spoilage micro-organisms and shelf-life of pork stored under air and modified atmosphere at −2, 4 and 10 °C. Food Microbiol..

[11] Liu F., Guo Y.Z., Li Y.F. (2006). Interactions of microorganisms during natural spoilage of pork at 5 °C. J. Food Eng..

[12] FSIS U (1996). Pathogen reduction: Hazard analysis and critical control point (HACCP) systems; final rule. Federal Register..

[13] Lagerstedt Å., Enfält L., Johansson L., Lundström K. (2008). Effect of freezing on sensory quality, shear force and water loss in beef *M. longissimus dorsi*. Meat Sci..

[14] Farouk M.M., Wieliczko K.J., Merts I. (2003). Ultra-fast freezing and low storage temperatures are not necessary to maintain the functional properties of manufacturing beef. Meat Sci..

[15] Wheeler T.L., Miller R.K., Savell J.W., Cross H.R. (1990). Palatability of chilled and frozen beef steaks. J. Food Sci..

[16] Franco C.M., Quinto E.J., Fente C., Rodriguez-Otero J.L., Dominguez L., Cepeda A. (1995). Determination of the principal sources of *Listeria* spp. contamination in poultry meat and a poultry processing plant. J. Food Prot..

[17] Leygonie C., Britz T.J., Hoffman L.C. (2012). Impact of freezing and thawing on the quality of meat: Review. Meat Sci..

[18] Jouki M., Khazaei N. (2011). Effects of storage time on some characteristics of packed camel meat in low temperature. Int. J. Anim. Vet Adv..

[19] Vieira C., Diaz M.T., Martínez B., García-Cachán M.D. (2009). Effect of frozen storage conditions (temperature and length of storage) on microbiological and sensory quality of rustic crossbred beef at different states of ageing. Meat Sci..

[20] Ngapo T.M., Babare I.H., Reynolds J., Mawson R.F. (1999). Freezing and thawing rate effects on drip loss from samples of pork. Meat Sci..

[21] Yang C.C., Chen T.C. (1993). Effects of refrigerated storage, pH adjustment, and marinade on color of raw and microwave cooked chicken meat. Poult Sci..

[22] MS 1500:2009 Malaysian Department of Standards (DOS) (2009). Halal Food-Production, Preparation, Handling and Storage-General Guidelines (Second Revision).

[23] Honikel K.O. (1998). Reference methods for the assessment of physical characteristics of meat. Meat Sci..

[24] Nakyinsige K., Fatimah A.B., Aghwan Z.A., Zulkifli I., Goh Y.M., Sazili A.Q. (2014). Bleeding efficiency and meat oxidative stability and microbiological quality of New Zealand white rabbits subjected to halal slaughter without stunning and gas stun-killing. Asian-Australas J. Anim. Sci..

[25] Hunter R.S., Harold R.W. (1987). The Measurement of Appearance.

[26] Bendall J.R. (1975). Cold-contracture and ATP-turnover in the red and white musculature of the pig, post mortem. J. Sci. Food Agric..

[27] Sabow A.B., Zulkifli I., Goh Y.M., Ab Kadir M.Z.A., Kaka U., Imlan J.C., Abubakar A.A., Adeyemi K.D., Sazili A.Q. (2016). Bleeding efficiency, microbiological quality and oxidative stability of meat from goats subjected to slaughter without stunning in comparison with different methods of pre-slaughter electrical stunning. PLoS ONE.

[28] Offer G., Trinick J. (1983). On the mechanism of water holding in meat: The swelling and shrinking of myofibrils. Meat Sci..

[29] Farouk M.M., Al-Mazeedi H.M., Sabow A.B., Bekhit A.E.D., Adeyemi K.D., Sazili A.Q., Ghani A. (2014). Halal and kosher slaughter methods and meat quality: A review. Meat Sci..

[30] Yu L.H., Lee E.S., Jeong J.Y., Paik H.D., Choi J.H., Kim C.J. (2005). Effects of thawing temperature on the physicochemical properties of pre-rigor frozen chicken breast and leg muscles. Meat Sci..

[31] Abdulla N.R., Mohd Zamri A.N., Sabow A.B., Kareem K.Y., Nurhazirah S., Ling F.H., Sazili A.Q., Loh T.C. (2017). Physico-chemical properties of breast muscle in broiler chickens fed probiotics, antibiotics or antibiotic–probiotic mix. J. Appl. Anim. Res..

[32] Muela E., Sañudo C., Campo M.M., Medel I., Beltrán J.A. (2010). Effect of freezing method and frozen storage duration on instrumental quality of lamb throughout display. Meat Sci..

[33] Swatland H.J. (2008). How pH causes paleness or darkness in chicken breast meat. Meat Sci..

[34] Bekhit A.E.D., Faustman C. (2005). Metmyoglobin reducing activity. Meat Sci..

[35] Kim G., Jeong J.Y., Hur S.J., Yang H.S., Jeon J.T., Joo S.T. (2010). The relationship between meat color (CIE *L** and *a**), myoglobin content, and their influence on muscle fiber characteristics and pork quality. Korean J. Food Sci. An..

[36] Abdallah M.B., Marchello J.A., Ahmad H.A. (1999). Effect of freezing and microbial growth on myoglobin derivatives of beef. J. Agric. Food Chem..

[37] Allen C.D., Russell S.M., Fletcher D.L. (1997). The relationship of broiler breast meat color and pH to shelf-life and odor development. Poult. Sci..

[38] Zhu L.G., Brewer M.S. (1998). Discoloration of fresh pork as related to muscle and display conditions. J. Food Sci..

[39] Leygonie C., Britz T.J., Hoffman L.C. (2011). Oxidative stability of previously frozen ostrich *Muscularis iliofibularis* packaged under different modified atmospheric conditions. Int. J. Food Sci..

[40] Kobayashi K.I., Matsui Y., Maebuchi Y., Toyota T., Nakauchi S. (2010). Near infrared spectroscopy and hyperspectral imaging for prediction and visualisation of fat and fatty acid content in intact raw beef cuts. J. Near Infrared Spec..

[41] Salwani M.S., Adeyemi K.D., Sarah S.A., Vejayan J., Zulkifli I., Sazili A.Q. (2015). Skeletal muscle proteome and meat quality of broiler chickens subjected to gas stunning prior slaughter or slaughtered without stunning. Cyta-J. Food.

[42] Devine C.E., Payne S.R., Peachey B.M., Lowe T.E., Ingram J.R., Cook C.J. (2002). High and low rigor temperature effects on sheep meat tenderness and ageing. Meat Sci..

[43] Farouk M.M., Wieliczko K.J. (2003). Effect of diet and fat content on the functional properties of thawed beef. Meat Sci..

[44] Shanks B.C., Wulf D.M., Maddock R.J. (2002). Technical note: The effect of freezing on Warner–Bratzler shear force values of beef *longissimus* steaks across several post mortem aging periods. J. Anim. Sci..

[45] Sabow A.B., Sazili A.Q., Zulkifli I., Goh Y.M., Kadir M.Z.A.A., Adeyemi K.D. (2015). Physico-chemical characteristics of L ongissimus lumborum muscle in goats subjected to halal slaughter and anesthesia (halothane) pre-slaughter. Anim. Sci. J..

[46] Djenane D., Aïder M., Yangüela J., Idir L., Gómez D., Roncalés P. (2012). Antioxidant and antibacterial effects of Lavandula and Mentha essential oils in minced beef inoculated with E. coli O157: H7 and S. aureus during storage at abuse refrigeration temperature. Meat Sci..

[47] Fung Y.C., Toldra F. (2010). Part 3: Microbial hazards in foods: Food-borne infections and intoxications. Handbook of Meat Processing.

